# SLOW WAVE SLEEP AND PAIN AFTER BEHAVIORAL INSOMNIA TREATMENT IN ADULTS OVER AGE 50 WITH KNEE OSTEOARTHRITIS

**DOI:** 10.1093/geroni/igz038.175

**Published:** 2019-11-08

**Authors:** Kathi L Heffner, Kathi L Heffner, Christopher R France, Wilfred R Pigeon

**Affiliations:** 1 University of Rochester Medical Center, Rochester, New York, United States; 2 Ohio University, Athens, Ohio, United States

## Abstract

Sleep disturbance can aggravate pain, and we recently found that insomnia treatment improved osteoarthritis (OA) pain, lowered inflammation, and improved quality of life in middle-to-older aged adults. Inadequate slow wave sleep (SWS), known as deep or restorative sleep, can decline with aging and is linked to pain and inflammation. We examined how insomnia treatment affects SWS, and the relationship between SWS and pain. In a pilot trial, 33 adults, ages 51 to 74 years with OA-related knee pain and insomnia, were randomized to 6-session CBTi (n=16) or a weekly phone contact control group (n=17). The CBT-I group showed significantly more laboratory-measured SWS across a study night than controls after controlling for baseline SWS. Greater SWS intensity was associated with lower OA-related pain among the CBT-I group, but not among controls. These preliminary data suggest that behavioral sleep treatment may strengthen the beneficial influence of restorative sleep on pain.

